# Euchromatic Supernumerary Chromosomal Segments—Remnants of Ongoing Karyotype Restructuring in the *Prospero autumnale* Complex?

**DOI:** 10.3390/genes9100468

**Published:** 2018-09-27

**Authors:** Tae-Soo Jang, John S. Parker, Hanna Weiss-Schneeweiss

**Affiliations:** 1 Department of Botany and Biodiversity Research, University of Vienna, A-1030 Vienna, Austria; 2 Department of Biology, College of Bioscience and Biotechnology, Chungnam National University, Daejeon 34134, Korea; jangts@cnu.ac.kr; 3 Cambridge University Botanic Garden, Cambridge CB2 1JF, UK; jsp25@cam.ac.uk

**Keywords:** FISH (fluorescence in situ hybridisation), GISH (genomic in situ hybridisation), *Prospero autumnale* complex, supernumerary chromosomal segments (SCS) evolution, tandem repeats

## Abstract

Supernumerary chromosomal segments (SCSs) represent additional chromosomal material that, unlike B chromosomes, is attached to the standard chromosome complement. The *Prospero autumnale* complex (Hyacinthaceae) is polymorphic for euchromatic large terminal SCSs located on the short arm of chromosome 1 in diploid cytotypes AA and B^7^B^7^, and tetraploid AAB^7^B^7^ and B^6^B^6^B^7^B^7^, in addition to on the short arm of chromosome 4 in polyploid B^7^B^7^B^7^B^7^ and B^7^B^7^B^7^B^7^B^7^B^7^ cytotypes. The genomic composition and evolutionary relationships among these SCSs have been assessed using fluorescence in situ hybridisation (FISH) with 5S and 35S ribosomal DNAs (rDNAs), satellite DNA *PaB6*, and a vertebrate-type telomeric repeat TTAGGG. Neither of the rDNA repeats were detected in SCSs, but most contained *PaB6* and telomeric repeats, although these never spanned whole SCSs. Genomic in situ hybridisation (GISH) using A, B^6^, and B^7^ diploid genomic parental DNAs as probes revealed the consistently higher genomic affinity of SCSs in diploid hybrid B^6^B^7^ and allopolyploids AAB^7^B^7^ and B^6^B^6^B^7^B^7^ to genomic DNA of the B^7^ diploid cytotype. GISH results suggest a possible early origin of SCSs, especially that on chromosome 1, as by-products of the extensive genome restructuring within a putative ancestral *P. autumnale* B^7^ genome, predating the complex diversification at the diploid level and perhaps linked to B-chromosome evolution.

## 1. Introduction

Karyotypes of plants and animals sometimes carry supernumerary genetic material, either in the form of B-chromosomes (Bs) or physically integrated into the standard chromosome complement as supernumerary chromosomal segments (SCSs) [1,2]. SCSs in plants are widespread [3], but are most frequently found in monocotyledons [4,5,6,7,8,9,10]. They are usually heterochromatic, but euchromatic segments also occur [11,12]. SCSs are frequently located terminally, and are thus seen at meiosis as heteromorphic bivalents resulting from chiasma formation proximal to SCSs [3,5,7,13]. SCSs usually behave as selfish genetic elements [9], but their inheritance can sometimes be Mendelian [14], and both of these patterns may be found within a single species [3].

A model system for investigating SCSs is provided by the *Prospero autumnale* complex (L.) Speta (Hyacinthaceae) [6,12]. This species complex has four evolutionarily and phylogenetically well-characterised and genomically distinct diploid cytotypes AA, B^5^B^5^, B^6^B^6^, and B^7^B^7^, each with unique combinations of genome size, base chromosome number (*x* = 5, 6 and 7), and repetitive DNA amounts and distribution [15]. Such chromosome number variation results from the relatively high rate of genome rearrangements in *Prospero*, involving translocations and inversions [6,15]. Hybridisation and polyploidisation of three of these diploid cytotypes (AA, B^6^B^6^, and B^7^B^7^) have given rise to further polyploid cytotypes, including autopolyploids of genome B^7^ (*x* = 7) [16,17,18,19,20] and two classes of allopolyploids, of A/B^7^ (both *x* = 7) and of B^6^/B^7^ genome composition [20,21,22,23]. Both B chromosomes (Bs) and SCSs have been reported in the three diploid cytotypes AA, B^6^B^6^, and B^7^B^7^ [15], and in various polyploids [6,17,21,24]. SCSs in *P. autumnale* are preferentially located terminally on the short arms of chromosomes 1 and 4 in the A and B^7^ genomes and are frequently quite large. Thus, the SCS on chromosome 1 may be nearly double the length of the chromosome. Remarkably, no phenotypic effects have been ascribed to the presence of these massive elements [16,23], although SCSs are geographically widespread and reach polymorphic proportions in many populations [6].

The origins of SCSs, particularly of the euchromatic ones, are obscure [2,3]. They may result from amplification of part of the genome, such as tandem repeats, especially satellite DNAs [25], or may represent inessential chromosome blocks generated by karyotype rearrangements and retained during chromosomal evolution [11,26,27]. In this paper, the structure, repeat composition, and origins of SCSs have been addressed in the *P. autumnale* complex using molecular cytological tools based on FISH (fluorescence in situ hybridisation) and GISH (genomic in situ hybridisation). In particular, the distribution patterns of tandem repeats (rRNA genes, satellite DNA, telomeric sequences) within SCSs have been established in a range of diploid and polyploid cytotypes to assess whether SCSs reflect single or multiple origins and what their relationships to specific genomes or chromosomal locations might be.

## 2. Materials and Methods

### 2.1. Plant Materials

Plants for cytogenetic analysis were collected from natural populations across the Mediterranean basin [15,20] and grown in the Botanical Garden of the University of Vienna. Ten diploid and nine polyploid (4*x* and 6*x*) plants carrying SCSs were studied and their collection details are listed in Table 1.

For cytological investigations, root meristems were pretreated with a solution of 0.05% colchicine (Sigma Aldrich, Vienna, Austria) for 4.5 h at room temperature, fixed in ethanol: acetic acid (3:1) for at least 3 h at room temperature, and stored at −20 °C until use.

### 2.2. Karyotyping and FISH (Fluorescence In Situ Hybridisation with 5S & 35S Ribosomal DNAs, Vertebrate-Type Telomeric Repeats, and Satellite DNA *PaB6*)

Chromosome numbers and karyotypes were analysed as described by Jang et al. [15,20] using standard Feulgen staining. Chromosomal spreads for FISH and GISH were prepared by enzymatic digestion and squashing, as described in Jang et al. [15,20].

Probes used for FISH were: satellite DNA *PaB6* isolated from the B^6^ genome in plasmid pGEM-T easy [28], 35S rDNA (18S/25S rDNA) from *Arabidopsis thaliana* in plasmid pSK+, and 5S rDNA from *Melampodium montanum* (Asteraceae) in plasmid pGEM-T easy, labeled with biotin or digoxygenin (Roche, Vienna, Austria). Probes were labeled either directly by PCR (5S rDNA and satellite DNA *PaB6*) or using a nick translation kit (35S rDNA; Roche). A commercially available, directly Cy3-labelled PNA (peptide nucleic acid) probe to vertebrate telomeric sequences (CCCTAA)_3_ (Dako, Glostrup, Denmark) was used as described in [10]. FISH was performed as described in Jang et al. [15,20].

### 2.3. GISH (Genomic In Situ Hybridisation)

GISH has been performed in one diploid hybrid individual B^6^B^7^ (H258) and four allopolyploid individuals, two of AAB^7^B^7^ (H110–1 & 2) and two of B^6^B^6^B^7^B^7^ (H574–1 & 2) composition, using labelled parental diploid genomic DNA as probes [24,29]. Total genomic DNA from diploid cytotypes AA, B^6^B^6^, and B^7^B^7^ was isolated using the CTAB (Cetyltrimethylammonium Bromide) method [29] and sheared at 98 °C for 5 min. Approximately 1 μg of genomic DNA of each cytotype was labeled using either digoxigenin or a biotin nick translation kit (Roche). GISH was carried out following the method of [29].

All preparations after FISH and GISH were analysed with an AxioImager M2 epifluorescent microscope (Carl Zeiss, Vienna, Austria), and images were captured with a CCD camera and processed using AxioVision ver. 4.8 (Carl Zeiss) with only those functions that apply to all pixels of the image equally.

## 3. Results and Discussion

### 3.1. Karyotype Structure and Localisation of Supernumerary Chromosomal Segments

**Figure 1:**
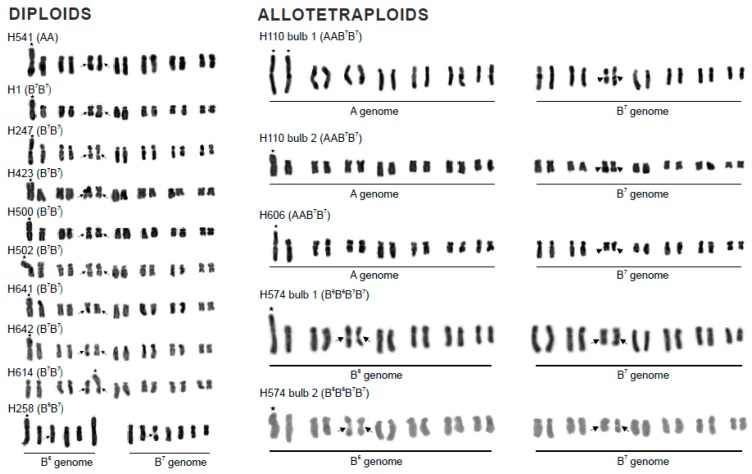

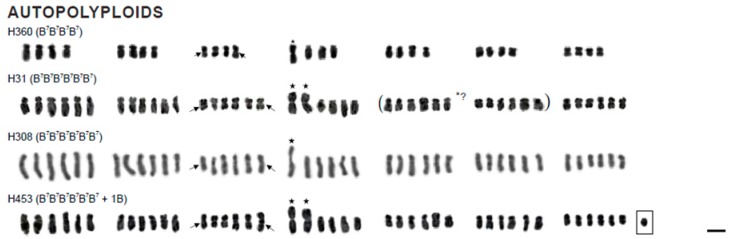
Karyotypes of *P. autumnale* individuals representing diploid and polyploid cytotypes of the *P. autumnale* complex carrying SCSs. Stars and arrows indicate SCSs and nucleolar organizer regions (NORs), respectively. Individual H31: Question mark above the bracket indicates chromosomes that potentially carry the small third SCS, as identified by FISH (fluorescence in situ hybridization) with the *PaB6* satellite DNA (see Figure 2p). Inset shows B-chromosome. Bar = 5 μm.

Nineteen SCS-carrying plants of the *P. autumnale* complex have been analysed (Table 1; Figure 1): ten diploids (one AA, eight B^7^B^7^, and one diploid hybrid B^6^B^7^; Figure 1) and nine polyploids (two B^6^/B^7^ allotetraploids, three A/B^7^ allotetraploids, and four B^7^ autopolyploids, one tetra- and three hexaploids; Figure 1). All SCSs are located distally, most frequently on chromosomes 1 and 4, and occasionally on chromosome 3 or either 5 or 6 (it is not possible to unambiguously assign this SCS to a specific chromosome; Figure 1). Chromosomes 1, 3, and 4 carry SCSs on their short arms and are thus are more symmetrical than their standard counterparts, while chromosome 5/6 carries an SCS on the long arm and is thus more asymmetric (Figure 1). The SCSs are massive blocks of chromatin and their length varies from 1.92 µm to 3.85 µm, representing a 21.4% to 36.4% increase in chromosome length, as found previously (Table 1) [6,16,21]. The SCS on chromosome 1 in the allotetraploid B^6^B^6^B^7^B^7^ is reported here for the first time (Figure 1).

The SCSs of chromosome 1 are remarkably constant in size across the distribution range of *P. autumnale* and also across ploidy levels. Thus, the SCS in B^7^B^7^ diploids has a length of about 2 μm in plants from Greece, Montenegro, and Spain, while that in the A genome is about 2.75 μm in length in AA diploids and in A/B^7^ allotetraploids (Table 1). The same constancy is not found in SCSs attached to other chromosomes.

### 3.2. Tandem Repeats in Supernumerary Chromosomal Segments

**Figure 2:**
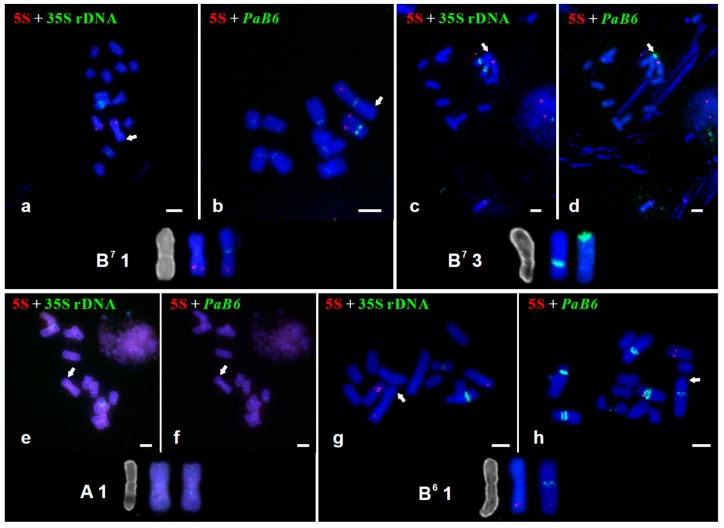

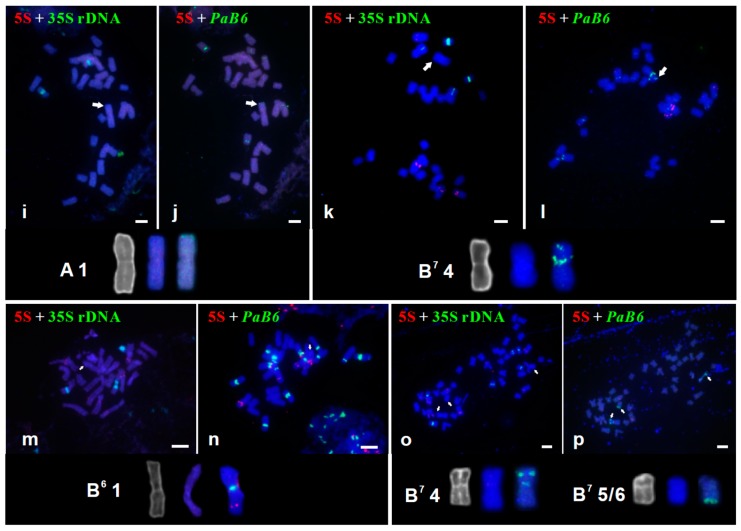
Localisation of 5S and 35S rDNAs and satellite DNA *PaB6* in diploid and polyploid cytotypes of the *P. autumnale* complex carrying SCSs. (**a**,**b**) B^7^B^7^ (H1); (**c**,**d**) B^7^B^7^ (H614); (**e**,**f**) AA (H541); (**g**,**h**) B^6^B^7^ (H258); (**I**,**j**) AAB^7^B^7^ (H110–2); (**k**,**l**) B^7^B^7^B^7^B^7^ (H360); (**m**,**n**) B^6^B^6^B^7^B^7^ (H574–1); (**o**,**p**) B^7^B^7^B^7^B^7^B^7^B^7^ (H31). Arrows indicate SCSs. Bar = 5 μm.

Four types of tandem repeats–35S rDNA, 5S rDNA, satellite DNA *PaB6*, and telomeric repeats–have been used as FISH probes on standard and SCS-carrying chromosomes of *P. autumnale* cytotypes (Figure 2). No signals for 35 or 5S rDNAs were detected within SCSs (Figure 2).

In standard karyotypes of diploids of *P. autumnale*, satellite DNA *PaB6* is located in pericentromeric regions of at least one chromosome (AA) and up to all chromosomes (B^6^B^6^, B^7^B^7^) [28]. By contrast, in SCSs, it is located terminally on chromosome 1 of B^7^B^7^ (2 of 7 plants), chromosome 1A of AAB^7^B^7^, chromosome 3 of B^7^B^7^, chromosomes 4 of B^7^B^7^B^7^B^7^ and B^7^B^7^B^7^B^7^B^7^B^7^, and chromosome 5/6 of B^7^B^7^B^7^B^7^B^7^B^7^ individuals (Figure 1 and Figure 2). No *PaB6* signals were detected on the SCSs of the AA diploid, of the B^6^B^7^ diploid hybrid, and of the five remaining B^7^B^7^ plants (Figure 2).

In the *P. autumnale* complex, vertebrate-type telomeric signals are found at the termini of both arms of all standard chromosomes (Figure S1) [28], and also in pericentromeric regions, coinciding with satellite DNA *PaB6* as its monomers contain a few full repeats of telomeric sequence TTAGGG (Figure S1b,c,e,f). Telomeric repeat signals in telomeric positions in standard chromosomes (i.e., lacking SCSs) were often very weak. In SCSs, slight amplification of telomeric repeats was detected in SCSs on chromosomes 1 of some B^7^ diploids (Figure S1d) and AAB^7^B^7^ allotetraploids (Figure S1b,c), always subterminally and coincident with amplification of satellite DNA *PaB6*. 

### 3.3. Genomic DNA Affinities of the Supernumerary Chromosomal Segments (Genomic In Situ Hybridisation)

**Figure 3:**
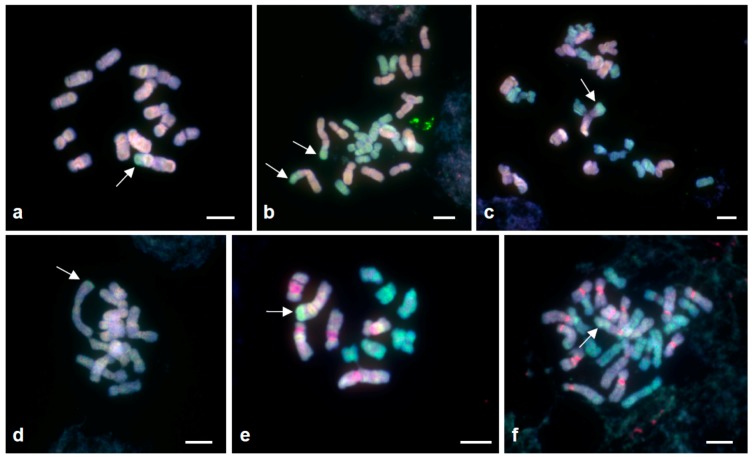
GISH in the *P. autumnale* complex. A–D: Localisation of A (red) and B^7^ (green) genomic DNA, (**a**): H541 (cytotype AA, 2*n* = 2*x* = 14, with one SCS), (**b**): H110–1 (cytotype AAB^7^B^7^, 2*n* = 4*x* = 28, with two SCSs), (**c**): H110–2 (AAB^7^B^7^, 2*n* = 4*x* = 28, with one SCS), (**d**): H641 (B^7^B^7^, 2*n* = 2*x* = 14, with one SCS), (**e**,**f**): Localization of B^6^ (red) and B^7^ (green) parental genomic DNAs in (**e**): H258 (B^6^B^7^ hybrid 2*n* = 2*x* = 13 with one SCS), (**f**): H574–1 (B^6^B^6^B^7^B^7^, 2*n* = 4*x* = 28, with one SCS). Arrows indicate SCSs. Bar = 5 μm.

The relationships of SCSs to parental genomes can be established using formamide-free GISH [20,29] in the diploid hybrid B^6^B^7^ (Figure 3e), and in the allotetraploids B^6^B^6^B^7^B^7^ (Figure 3f) and AAB^7^B^7^ (Figure 3b,c). In all cases, the SCSs have a higher affinity for the B^7^ genomic probe than for either of the other two parental genomic probes (A or B^6^ genomes; Figure 3b,c,e,f).

Remarkably, the SCS of chromosome 1 of the AA diploid shows hybridisation with B^7^ genomic DNA (Figure 3a). As expected, no discrimination is shown by the chromosome 1 SCS in B^7^B^7^ diploid plants used as the control (Figure 3d). A possible explanation for this is hybridisation at the diploid level between AA and B^7^B^7^ plants. Recombination between the SCSs-carrying B^7^1 chromosome and an A1 chromosome may have occurred in the diploid background, allowing the transfer of this SCS from B^7^1 to A1 (A/B chiasma formation has been seen in AB^7^B^7^ triploids) [30]. Subsequent recurrent backcrossing to AA would restore the AA complement, but with the addition of an SCS derived from B^7^1.

**Figure 4:**
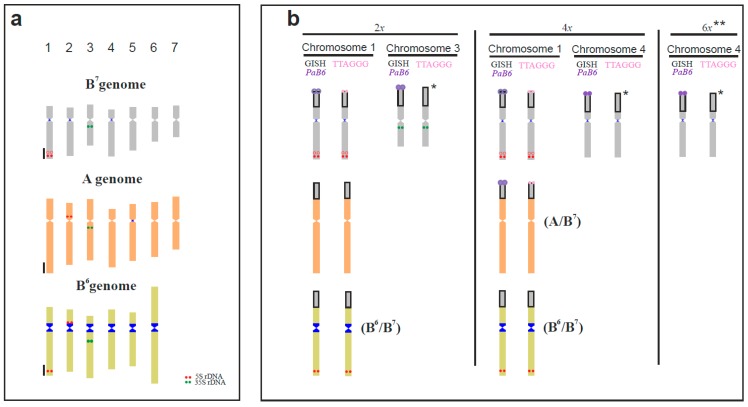
Summary of all SCSs types present in diploid and polyploid individuals of *P. autumnale*: (**a**) ideograms of standard diploid cytotypes, B^7^B^7^ (gray), AA (orange), and B^6^B^6^ (lime) with 5S rDNA, 35S rDNA, and *PaB6* loci indicated (modified from Emadzade et al. 2014); (**b**) genomic affinity assessed by GISH and distribution of repetitive DNAs (rDNAs, satDNA *PaB6*, and vertebrate-type telomeric sequence TTAGGG) in SCSs in analysed diploids (2*x*), tetraploids (4*x*), and hexaploids (6*x*). *—not analysed; **—chromosome 5/6 is not indicated due to our inability of assigning the SCS to chromosome 5 or 6.

The current study by means of FISH and GISH indicates that, in the complex evolutionary system of *P. autumnale,* the origin of at least some SCSs can be traced back to the B^7^ genome (Figure 4). Previously, it was hypothesized that the ancestral karyotype of the complex closely resembled that of B^7^B^7^ [15,28]. From this diploid complement, both B^6^B^6^ and B^5^B^5^ cytotypes have been derived by one and two independent fusions, respectively [15]. The generation of the SCSs, so remarkably prevalent in the complex, may thus be an outcome of chromosomal rearrangements associated with the generation of new cytotypes with new base chromosome numbers and karyotype structures. At least some of these SCSs might be quite old, their origin preceding the diversification of the extant diploid cytotypes. Extensive chromosomal rearrangements in *P. autumnale* have also been proposed to be the most likely reason for its extraordinary variability (both structural and genomic) and the ongoing origin of B chromosomes [24]. It is possible that the putative ancestral SCS of chromosome 1 has originated from a B chromosome that translocated to chromosome 1 early in the diversification of the *P. autumnale* complex. Other SCSs may be of a different age and their formation might also be ongoing.

We may postulate, then, that all of the SCSs of chromosomes 1 found in *P. autumnale* derived from a single event, and the presence of a B^7^-like SCS on the short arm of chromosome 1 of the A-genome supports this contention. It is clear, however, that the SCSs now differ in their molecular structure between chromosomes and between cytotypes, as we have demonstrated with repetitive DNA probes. This may simply reflect the profound genomic changes that have swept through the complex since its origin [15,28]. This common descent of SCSs, however, should be further explored through GISH studies and molecular analyses of many more plants, making use of the extraordinary levels of chromosomal polymorphism found in natural populations of *P. autumnale* [16,20,21,23].

## Figures and Tables

**Table 1:** List of plant material of the *Prospero autumnale* complex studied with voucher information and chromosome numbers. The chromosomal location, genomic affinity (as established using GISH), and length of supernumerary chromosomal segments (SCSs) are indicated.

Cytotype (Accession Number)	Locality; Collector	2*n*	Genomic Location/Genomic Affinity of SCSs	SCS Length (µm)	Proportion of SCS (%) in the Chromosome ^†^	Figure No.
Diploids with SCSs						
AA (H541)	Spain, Huelva; J. Parker	14	A 1/genome B^7^	2.67	28.8	Figure 1, Figure 2e,f, Figure 3a and Figure S1a
B^7^B^7^ (H1)	Greece, Rhodos; F. Speta	14	B^7^ 1	2.02	28.8	Figure 1 and Figure 2a,b
B^7^B^7^ (H247)	Greece, Crete; F. Speta	14	B^7^ 1	2.02	24.5	Figure 1
B^7^B^7^ (H423)	Montenegro; F. Speta	14	B^7^ 1	2.07	31.3	Figure 1
B^7^B^7^ (H500)	Greece, Crete; F. Speta	14	B^7^ 1	2.02	30.6	Figure 1
B^7^B^7^ (H502)	Greece, Crete; F. Speta	14	B^7^ 1	2.07	33.3	Figure 1
B^7^B^7^ (H614)	Israel, HaCarmel Park; J. Parker	14	B^7^ 3	2.29	36.4	Figure 1 and Figure 2c,d
B^7^B^7^ (H641)	Spain; J. Parker	14	B^7^ 1	1.98	32.0	Figure 1, Figure 3d and Figure S1d
B^7^B^7^ (H642)	Spain; J. Parker	14	B^7^ 1	2.07	27.8	Figure 1
B^6^B^7^ (H258)	Greece, Crete; F. Speta	13	B^6^ 1	2.11	23.4	Figure 1, Figure 2g,h,e and Figure S1e
Polyploids with SCSs						
AAB^7^B^7^ (H110–1)	Portugal, Cheleiros; F. Speta	28	A 1 *	3.16	21.4	Figure 1, Figure 3b and Figure S1b
AAB^7^B^7^ (H110–2)	Portugal, Cheleiros; F. Speta	28	A 1	2.63	33.3	Figure 1, Figure 2i,j, Figure 3c and Figure S1c
AAB^7^B^7^ (H606)	Portugal, Castro Marin; J. Parker	28	A 1	2.89	26.2	Figure 1
B^6^B^6^B^7^B^7^ (H574–1)	Greece, Naxos; F. Speta	28	B^6^ 1	3.85	27.3	Figure 1, Figure 2m,n, Figure 3f and Figure S1f
B^6^B^6^B^7^B^7^ (H574–2)	Greece, Naxos; F. Speta	28	B^6^ 1	3.21	31.3	Figure 1
B^7^B^7^B^7^B^7^ (H360)	Greece, Kos; F. Speta	28	B^7^ 4	1.94	35.3	Figure 1 and Figure 2k,l
B^7^B^7^B^7^B^7^B^7^B^7^ (H31)	No information; F. Speta	42	B^7^ 4 * and (5 or 6) ^§^	1.92 ^§^	33.3 ^§^	Figure 1 and Figure 2o,p
B^7^B^7^B^7^B^7^B^7^B^7^ (H308)	Croatia, Solta; F. Speta	42	B^7^ 4	2.88	34.6	Figure 1
B^7^B^7^B^7^B^7^B^7^B^7^ (H453)	Croatia, Mosor; F. Speta	42 + 1B	B^7^ 4 *	2.56	33.3	Figure 1

*: Homozygous SCS; ^†^: Proportion of SCSs within the chromosomes carrying them, ^§^: Only SCSs of chromosomes 4 were measured because the other SCSs could not be unambiguously assigned to chromosome 5 or 6. GISH: Genomic in situ hybridization.

## Supplementary Materials

The following are available online at http://www.mdpi.com/2073-4425/9/10/468/s1. Figure S1: Localisation of Cy3-labelled telomeric (TTAGGG)n repeats in supernumerary chromosomal segments (SCSs) (arrowed) in diploid and polyploid cytotypes of the *Prospero autumnale* complex. (a) H541 (cytotype AA with one SCS), (b) H110–1 (cytotype AAB7B7 with two SCSs), (c) H110–2 (cytotype AAB7B7 with one SCS), (d) H641 (cytotype B7B7 with one SCS), (e) H258 (B6B7 hybrid with one SCS), (f) H574–1 (cytotype B6B6B7B7 with one SCS).
