# Supplementary material for: Euchromatic Supernumerary Chromosomal Segments—Remnants of Ongoing Karyotype Restructuring in the Prospero autumnale Complex?
Source: Genes (Basel). 2018 Sep 27;9(10):468. doi: 10.3390/genes9100468 (PMC6210179; doi:10.3390/genes9100468)
Supplement: Supplementary file 1 [file genes-09-00468-s001.pdf]

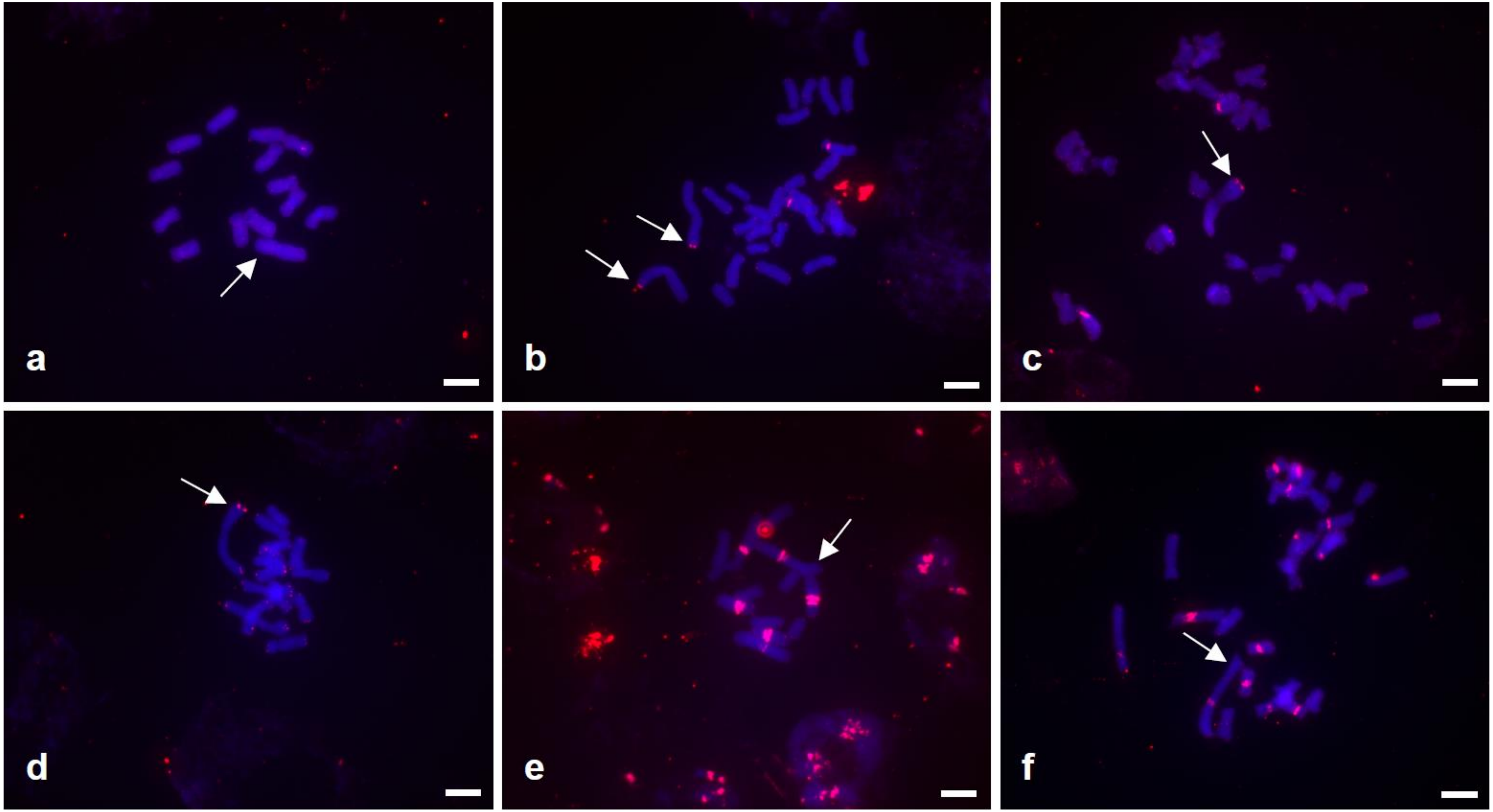

Figure S1. Localisation of Cy3-labelled telomeric (TTAGGG)n repeats in supernumerary chromosomal segments (SCSs) (arrowed) in diploid and polyploid cytotypes of the *Prospero autumnale* complex. **(a)** H541 (cytotype AA with one SCS), **(b)** H110–1 (cytotype AAB^7B^7 with two SCSs), **(c)** H110–2 (cytotype AAB^7B^7 with one SCS), **(d)** H641 (cytotype B^7B^7 with one SCS), **(e)** H258 (B^6B^7 hybrid with one SCS), **(f)** H574–1 (cytotype $B^6B^6B^7B^7$ with one SCS). Bar = 5 μm.
